# One health, many interpretations: vaccinating risk groups against H5 avian influenza in Finland

**DOI:** 10.2807/1560-7917.ES.2024.29.25.2400383

**Published:** 2024-06-20

**Authors:** Hanna Nohynek, Otto Matias Helve

**Affiliations:** 1 Department of Health Security, Finnish Institute for Health and Welfare (THL), Helsinki, Finland

**Keywords:** Finland, viral infections, avian influenza, influenza virus, vaccines and immunisation, H5N1

Since 2020, highly pathogenic avian influenza (HPAI) A(H5N1) has caused widespread mortality in both wild and domestic birds in Europe and several consequent spillover events to mammals [1]. Outbreaks in fur farms in Europe and dairy cattle in North America serve as very recent examples of the unpredictable nature of the virus.

In July 2023, an HPAI A(H5N1) outbreak caused by clade 2.3.4.4b genotype BB was detected in fur farms in Finland [2]. Kareinen et al. describe in this issue of *Eurosurveillance* the epidemiological, pathological and genomic investigations of the outbreak [3]. Intensified surveillance activities identified 27 RT-PCR A(H5N1)-positive fur farms in western Finland. The outbreak was contained by the end of the year and affected nearly half a million farmed fur animals, mainly foxes and minks. Whole genome sequencing was carried out on outbreak viruses and shared as soon as available with the scientific community through the GISAID EpiFlu database. Phylogenetic analyses indicated several initial introductions of genotype BB clade 2.3.4.4b viruses through large-scale exposure of fur animals to infected wild birds. The genomic analyses further revealed several mutations indicative of early mammalian adaptation of the viruses in their mammalian hosts.

Animals in the affected farms were eventually culled [4]. The impact of culling 485,000 animals on a total of 72 farms was estimated by the Finnish Food Authority to have resulted in compensation for lost income of 50.7 million EUR to the owners of the affected farms [5]. The potentially relevant global health and economic consequences led to public discussion on the need to stop fur farming in Finland altogether, as was done in Denmark when during the COVID-19 pandemic farmed minks were infected by severe acute respiratory syndrome coronavirus 2 (SARS CoV-2) [6]. Recognising the constitutional rights of the fur farmers to their livelihood [7], the Finnish government instead focused on providing extensive advice to the fur farms how to protect farmed animals from getting into contact with wild birds. The public health authorities had a major role in the outbreak management due to its possible significant public and global health impact. The epidemiological data were constantly evaluated from a human health perspective and provided the bases for recommendations for the general population, healthcare providers, and other concerned parties. Also, viral evolution was monitored by the Finnish Food Authority to assess the potential human health impact of the mutations emerging in the virus during the fur farm epidemic. Whole genome sequencing and genomic analyses revealed several mutations indicative of an early mammalian adaptation of the viruses isolated during the fur farm outbreak in Finland in 2023 [2]. As a consequence, and in addition to several nonpharmaceutical measures, vaccines were considered as a tool to prevent severe human illness, as well as to minimise the risk of reassortment between seasonal and avian influenza, an event that may result in variants with novel properties and increased transmission potential in the human population.

Already during spring 2023, when the European and global HPAI epidemic became obvious [1], the decision was made that those at increased risk for avian influenza should be offered a new adapted avian influenza vaccine as soon as one received marketing authorisation from the European Medical Agency (EMA) [8]. The rationale was the precautionary principle, i.e. to provide protection via immunisation to enable continued fur farming in Finland. The target groups were identified in collaboration with the Finnish Food Authority, Ministry of Social Affairs and Health and the Finnish Institute for Health and Welfare (THL) (Box).

Box **Risk groups to whom the Finnish national public health institute recommends vaccination with the MF59-adjuvanted avian influenza vaccine**

- Persons in contact with farmed fur animals;
- Persons in contact with poultry;
- Persons handling sick or dead animals or cleaning the related facilities;
- Persons in charge of ringing birds;
- Person taking care of birds in animal care facilities;
- Persons working with birds in bird or livestock farms;
- Veterinarians working in the public sector;
- Laboratory personnel working with testing of avian influenza;
- Close contacts of confirmed or suspected human avian influenza cases.

The rationale is to protect the individual against serious forms of avian influenza, and to avoid further mutations which might lead to increased human-to-human transmission.

In addition, the same groups, in absence of a tailored avian influenza vaccine, were offered a seasonal influenza vaccine during autumn 2023 [9]. This could provide cross protection against HPAI, and also slow down potential reassortment should a simultaneous infection of seasonal and HPAI occur in the same individual. Fur farming poses a continued risk of avian influenza spillover from wild animals to fur animals, thereby exposing individuals handling these animals to the virus. Since January 2024, stringent requirements for increased biosafety in fur farms have been mandated by the Ministry of Agriculture and Forestry [10]. However, the implementation of these measures is not assessed.

In relation to vaccination of humans, the European Commission’s Directorate-general (DG) Health Emergency Preparedness and Response (HERA) has made a joint procurement agreement with 15 countries and the vaccine manufacturer Seqirus [11] for an option of 40 million doses of the MF59-adjuvanted avian influenza vaccine, which is authorised by EMA under the brand name Zoonotic influenza vaccine, Seqirus [12]. The Zoonotic influenza vaccine Seqirus is based on A/Astrakhan/3212/2020 (H5N8)-like strain (CBER-RG8A) (clade 2.3.4.4b) and is expected to provide protection against clade 2.3.4.4b avian influenza viruses. It is highly likely that Finland will be the first country to start vaccinating in Europe. The United States where HPAI A(H5N1) was recently detected in dairy farm cattle, have reached an agreement to acquire 4.8 million doses of Zoonotic influenza vaccine for pandemic preparedness [13].

Surveillance remains intensified in wild animals and fur farms, and by June 2024, only one HPAI-positive wild bird has been detected in Finland in January 2024. Despite this present epidemiological situation, considering the previous events, the plan to vaccinate risk groups is materialising, and vaccinations are envisaged to start during the summer. The licensure took longer than the 100-day promise initiated by the Coalition for Epidemic Preparedness Innovations (CEPI) [14]. This is understandable since the outbreak is not a human pandemic at this stage. However, a spillover increases the potential pandemic threat when avian influenza transmits from one species to another, especially to a mammalian species.

The EMA’s guidance on the post-licensure safety surveillance of influenza vaccines lists three options: active, passive, and register-based [15]. In Finland, safety signal detection is the responsibility of the regulatory authority the Finnish Medicines Agency, while the national public health institute (THL) participates in signal validation and carrying out epidemiological studies to assess causality. Surveillance for the A(H5N8) vaccine safety will be passive. National registers using individual person identifier linkage can be used to carry out cohort or test-negative design studies [16] and THL will undertake a separate phase IV immunogenicity and safety study.

The THL aspires that individuals belonging to the target groups will accept the vaccine offered to them. Because of the holiday season and therefore limited primary healthcare resources, the summer period is a demanding time to implement any vaccination measures. The fact that an MF59-adjuvanted vaccine has never been used in the national immunisation programme in Finland before may raise questions among lay people and professionals alike even if MF59-adjuvanted vaccines have been safely given to millions during the influenza A(H1N1)pdm09 pandemic in 2009 and 2010 and seasonally in many high-income countries. In Finland, the pandemic influenza A(H1N1)pdm09 vaccine Pandemrix-related narcolepsy episode is still in the minds of many [17]. There are also questions on the degree and duration of protection the vaccine will provide.

The recent outbreak of HPAI H5N1 on Finnish fur farms raises larger concerns than just on its immediate impacts on human health. Finland is probably the first country in the world to initiate vaccinations against this particular HPAI, a measure that would probably not have been taken, had fur farming been discontinued. This approach, however, addresses only a small aspect of a broader issue that encompasses the vast array of human environmental impacts. This issue must be evaluated within a framework which considers the intricate interplay between the environment, animals, and humans [18]. Recognising this interconnectedness and the vast array of environmental impacts of human activity is crucial, and our protective measures should consider the overarching goal of maintaining and enhancing planetary health.

## Note

Hanna Nohynek and Otto Matias Helve are members of the THL NITAG Finnish National Immunization Technical Advisory Group (NITAG). Hanna Nohynek is chair of the World Health Organization Strategic Advisory Group of Experts on Immunization (WHO SAGE) and the Principal investigator of the immunological and safety study.
